# Transient islet-antigen 2 positivity at diagnosis of diabetes during COVID-19 infection in an adolescent with a *NEUROD1* variant: a case report

**DOI:** 10.1186/s13256-025-05327-7

**Published:** 2025-06-05

**Authors:** Jennifer K. Yee, Seerat Anand, Juan C. Sanabria, Catherine S. Mao

**Affiliations:** 1 Division of Endocrinology, Department of Pediatrics, Harbor-UCLA Medical Center, 1000 W. Carson St., Harbor Box 446, Torrance, CA 90509 USA; 2 Division of Endocrinology Fellowship Program, Department of Internal Medicine, Harbor-UCLA Medical Center, Torrance, CA USA; 3 Department of Family Medicine Residency Program, Harbor-UCLA Medical Center, Torrance, CA USA; 4 The Lundquist Institute of Biomedical Innovation at Harbor-UCLA Medical Center, Torrance, CA USA; 5 The David Geffen School of Medicine, University of California Los Angeles, Los Angeles, CA USA

**Keywords:** COVID-19, Autoimmunity, Maturity onset diabetes of the young (MODY), Case report

## Abstract

**Background:**

Increased incidence of type 1 and type 2 diabetes has been reported in association with the coronavirus disease 2019 pandemic. Little is known about the impact of coronavirus disease 2019 on the presentation of monogenic forms of diabetes. This case report describes diagnosis of the uncommon maturity onset diabetes of the young (neurogenic differentiation factor 1-maturity-onset diabetes of the young) after transient autoimmunity during coronavirus disease 2019 infection.

**Case presentation:**

A 19-year-old Mexican male with autism spectrum disorder was diagnosed with diabetes without ketoacidosis during metabolic screening laboratory testing. He tested positive for coronavirus disease 2019 with no respiratory symptoms. His hemoglobin A1c was 14.2%, and had elevated islet-antigen 2 antibody. His blood glucose responded rapidly to initial dietary reduction in carbohydrate intake without insulin initiation, with subsequent improvement in hemoglobin A1c to 7.4% 2 months later. Then, 2 years after diagnosis, his hemoglobin A1c was 6.0%, and repeat antibody testing was negative. He was taking lisinopril for microalbuminuria, but required no medications for glucose control. Genetic testing identified a variant of *NEUROD1* c.514C > T, p.(Leu172Phe). His mother who was subsequently diagnosed with diabetes, underwent targeted variant testing and was found to have the same neurogenic differentiation factor 1 variant.

**Conclusions:**

Coronavirus disease 2019 possibly triggered transient autoimmunity, leading to the diagnosis of neurogenic differentiation factor 1-maturity-onset diabetes of the young diabetes. This is also the first description of the clinical features associated with this particular neurogenic differentiation factor 1 variant.

## Introduction

The incidence of diabetes increased significantly among children during the coronavirus disease 2019 (COVID-19) pandemic [1], with studies reporting a 55% increase in type 1 diabetes after COVID-19 infection [2], and 77.2% overall increase of type 2 diabetes [3]. Much less is known about how COVID-19 impacted less common forms of diabetes mellitus. Maturity-onset diabetes of the young (MODY) encompasses a spectrum of rare, monogenic forms of diabetes, which account for 1–3% of cases diagnosed younger than 30 years of age. At least 14 genes have been associated with MODY, of which subtypes 1, 2, 3, and 5 are the most common and well-described [4]. Neurogenic differentiation factor 1 (NEUROD1)-MODY (MODY6) is a rare subtype caused by a mutation in the *NEUROD1 *gene on chromosome 2q32. Although first reported in 1999 [5], the literature on the clinical and genetic aspects of NEUROD1-MODY (MODY6) is limited, and it is relatively underexplored. Prior case reports and series describe considerable variations in age of onset, clinical features, and genetic mutations [6]. There is a literature gap regarding COVID-19 and MODY.

This report describes a family of Mexican descent affected by diabetes, with a *NEUROD1* variant whose clinical phenotype was previously not described in the literature. We describe the onset, clinical features, laboratory, and genetic findings in a young adult with this variant. Moreover, the presentation of diabetes occurred in the setting of a COVID-19 infection, with transient autoimmunity to islet-antigen 2 (IA-2). Given the unique aspects of this case, this report explores the intersection of NEUROD1-MODY (MODY6), autoimmunity, and COVID-19 infection.

## Case

A 19-year-old Mexican male with autism spectrum disorder was seen at a primary care visit with chief complaint of worsening hidradenitis suppurativa. Metabolic screening labs performed during the encounter (height 168.8 cm, weight 88.7 kg, and body mass index [BMI] 31.1 kg/m^2^) resulted with a serum glucose of 518 mg/dL, and he was referred to the emergency department for new onset diabetes evaluation. On further laboratory testing, his pH was 7.37, beta-hydroxybutyrate was 0.1 mmol/L, urinalysis was negative for ketones, and hemoglobin A1c (HbA1c) was 14.2%. He had a normal complete blood count (CBC), mild transaminitis (AST 56 U/L; ALT 59 U/L), and a low-density lipoprotein (LDL) level of 132 mg/dL. Severe acute respiratory syndrome coronavirus 2 (SARS CoV-2) testing was positive (polymerase chain reaction [PCR] test, Cepheid); however, he did not have any respiratory symptoms of COVID-19. His glucose level improved after intravenous fluid bolus. Hospitalization was not required due to clinical stability (lack of ketosis). Pediatric endocrinology was consulted to coordinate initiation of outpatient management. Laboratory tests were drawn for type 1 antibodies and C-peptide level.

The patient did not have acanthosis nigricans on exam. The parents are originally from the Zacatecas region of Mexico. His father was diagnosed with type 2 diabetes at the age of 57 years, and his mother was later diagnosed at the age of 59 years with type 2 diabetes. An older sister does not have diabetes. The grandparents did not have diabetes. His islet-antigen 2 (IA-2) antibody level was positive at 54.1 U/mL (< 5.4 U/mL), but glutamic acid decarboxylase 65 (GAD65, < 5 IU/mL) and insulin autoantibodies (IAA, < 0.4 U/mL) were undetectable. A C-peptide level was 1.80 ng/mL (0.8–3.85 ng/mL). The patient was started on an intermittently scanned continuous glucose monitor (CGM). Although insulin was prescribed, his blood glucose trend responded immediately to reduction in carbohydrate intake (glucose range 74–218 mg/dL by CGM) prior to the first dose. The patient was presumed to be entering a honeymoon phase, and insulin was not needed on the basis of the tight glucose trend.

The HbA1c decreased without insulin during the first year after diagnosis: 11.5% at 1 month, 7.4% at 2 months, and 5.7% at 1 year. Then, 2 years after diagnosis, the HbA1c was 6.0% on no medications, and the CGM readings showed a glucose average of 120 mg/dL, with 96% in target range (70–180 mg/dL), 3% above target, and 1% below target. The patient’s type 1 antibody levels were rechecked for staging of diabetes (GAD65, IA-2, IAA, ZnT8), and all were undetectable. He was started on lisinopril for microalbuminuria based on two elevated ratios of urine microalbumin/creatinine (40 and 54 mcg/mg; reference range < 30 mcg/mg Cr), with improvement to 31 mcg/mg during dose titration. He had no retinopathy on annual retinal imaging, and no peripheral neuropathy on foot exams. Due to the disappearance of the IA-2 antibodies, the patient underwent next generation sequence testing for maturity onset diabetes of the young (MODY) (Prevention Genetics, Marshfield, WI). The patient was identified to have a heterozygous variant of indeterminate significance for neurogenic differentiation factor 1 (*NEUROD1*) (NM_002500.4), c.514C > T, p.(Leu172Phe). The mother consented to targeted gene sequencing and was found to have the same heterozygous variant. The father was not available for testing. Written informed consent was obtained for this case report.

The variant was submitted through *in silico* prediction algorithms (by JY) to assess the potential to cause disease. The Mutation Taster prediction was “disease causing,” the PolyPhen-2 result was “probably damaging,” and the Functional Analysis through Hidden Markov Models (FATHMM) result indicated “damaging [7].” MutPred-2 reported a score of 0.648 (> 0.50 suggests pathogenicity), with “potential loss of proteolytic cleavage at S167,” with a probability of 0.11 and *p*-value of 0.05.

## Discussion

The incidence of diabetes increased globally during the pandemic years, including children and adolescents [1]. Moreover, the risk of onset of diabetes has been reported to be higher following COVID-19 infection, compared with non-exposure [8]. The published studies have typically focused on observations of type 1 and type 2 diabetes. This patient’s case is unique and interesting from several perspectives: (1) diagnosis of monogenic diabetes in association with COVID-19 infection, (2) identification of a variant of unknown significance for *NEUROD1*, a gene associated with NEUROD1-MODY (MODY6), and (3) presence of transient autoimmunity.

*NEUROD1*, located on chromosome 2q32, encodes a transcription factor expressed in the pancreatic islet cells, intestinal cells, and neurons of the central and peripheral nervous system [9]. NEUROD1 has a role in endocrine cell differentiation of the pancreas during organogenesis. NEUROD1 forms a heterodimer with E47 to bind to the insulin gene promoter. Homozygous mutations cause permanent neonatal diabetes and neurological abnormalities [6, 9], while heterozygous mutations have been reported to cause NEUROD1-MODY (MODY6). This subtype is rare compared with other MODY types, and much of the literature is based on case reports and case series [10]. Patients are reported to exhibit diabetes similar to type 2, with onset during adolescence to mature adulthood [11]. Incomplete penetrance among family members has been observed, including the neurologic features. Presentation with ketoacidosis has also been reported [11]. Among the published cases, the diabetes prevalence is twice that among female patients than male patients, and a greater proportion (85.7%) inherited the heterozygous mutations from the mother. Our patient’s presentation is consistent with diagnosis during late adolescence, and maternal inheritance of the variant.

Our patient’s specific variant was previously reported within a study of individuals with lipid metabolic disorders; however, no phenotype information was provided [12]. Therefore, this is the first report to describe clinical features associated with this variant. Many pathogenic variants have been reported in the basic helix–loop–helix (bHLH) region and the transactivation domains; however our patient’s variant occurs in a region between these domains. A mutation in the same region was reported [11] in a 20-year-old MODY6 patient c.470T > G, p.(Leu157Arg) who experienced ketoacidosis and evidence of nephropathy and required both insulin and oral medications for treatment. In contrast, our patient has maintained glycemic control without medications, and the mother was diagnosed with diabetes later at the age of 59 years, and is being treated with oral medications. Therefore, our patient’s particular *NEUROD1* variant appears to be associated with diabetes without ketoacidosis, and does not require insulin. However, he does demonstrate early onset of the renal microvascular complication of microalbuminuria. The treating clinicians speculate that autoimmunity and COVID-19 induced severe hyperglycemia transiently, prompting the genetic testing that led to identification of the *NEUROD1* variant.

The presence of transient autoimmunity with one type 1 diabetes antibody at the time of diagnosis was an unexpected finding, especially with lack of ketoacidosis despite extremely high glucose levels. Published research supports an increased risk of autoimmune disease including type 1 diabetes among COVID-19 patients [13]. Proposed mechanisms by which the coronavirus damages β-cells include triggering cell death, destruction of pancreatic β cells, and damage to β cells from surrounding infection. However, there is little data on outcomes that distinguish between long-term autoimmunity versus transient autoimmunity. There is published evidence to suggest that COVID-19 disease can be associated with transient hyperglycemia, but the autoimmune status is not clear in the study subjects [14]. In our patient with transient autoimmune diabetes and positivity in only one antibody, it is possible that COVID-19 was the primary trigger for transient autoimmunity and severe hyperglycemia. This case illustrates the need for better investigation into the role of autoimmunity in cases of COVID-19 associated dysglycemia, whether transient or permanent. Future studies may examine for correlations with severity of diabetes, and potential to predict intensity of management.

A potential relationship between type 1 autoimmunity and *NEUROD1* mutations has been explored in the literature. A polymorphism of the *NEUROD1* gene, leading to an Ala/Thr substitution in the NH_2_-terminus, was identified more frequently among Japanese individuals with acute onset type 1 diabetes compared with those without diabetes [15]. A recent case report in a child of Sri Lankan heritage [16] identified a heterozygous nonsense variant c.453G > A, p(Trp151*) in the bHLH region that was associated with mildly positive autoantibodies against insulin (2.9 U/mL; normal < 2.4 U/mL) and ZnT8 (25.1 U/mL; normal < 15 U/mL), but no high-risk haplotypes were found on HLA testing. The patient required both insulin and oral medications for blood glucose management. The co-authors proposed that the abnormal NEUROD1 protein could promote autoantibody production through endoplasmic reticulum (ER) stress and apoptosis. Another report [17] identified a family of Palestinian heritage with type 1 diabetes in three siblings associated with a *NEUROD1* homozygous variant c.590G > T, p(P197H) affecting the conserved transactivation domain of the protein. The heterozygous parents did not have diabetes. This case report continues to raise the question of whether the patient’s *NEUROD1* variant played any role in his autoimmunity, independent of COVID-19.

Strengths of this case report include genetic test results from both the son and the mother, and follow-up of glycemic control and antibody testing over time. A limitation is the lack of formal functional assays on this particular *NEUROD1* variant. However, *in silico* prediction algorithms were performed.

## Conclusion

In conclusion, our patient’s *NEUROD1* variant (c.514C > T, p.Leu172Phe) is associated with a mild phenotype of diabetes that does not require insulin and is not associated with ketoacidosis. He has the microvascular complication of microalbuminuria and should undergo screening for complications similar to type 2 diabetes patients. The patient, but not the mother, exhibits a neurologic feature of autism. COVID-19 infection may have triggered severe hyperglycemia through a transient autoimmune mechanism, and it remains an open question whether the genetic background contributed additional susceptibility to autoimmunity.

## Data Availability

Not applicable.

## References

[1] D’Souza D, Empringham J, Pechlivanoglou P, Uleryk EM, Cohen E, Shulman R. Incidence of diabetes in children and adolescents during the COVID-19 pandemic: a systematic review and meta-analysis. JAMA Netw Open. 2023;6(6): e2321281. 37389869 10.1001/jamanetworkopen.2023.21281 PMC10314307

[2] Weiss A, Donnachie E, Beyerlein A, Ziegler AG, Bonifacio E. Type 1 diabetes incidence and risk in children with a diagnosis of COVID-19. JAMA. 2023;329(23):2089–91. 37213115 10.1001/jama.2023.8674 PMC10203966

[3] Magge SN, Wolf RM, Pyle L, Brown EA, Benavides VC, Bianco ME, *et al*. The coronavirus disease 2019 pandemic is associated with a substantial rise in frequency and severity of presentation of youth-onset type 2 diabetes. J Pediatr. 2022;251:51-9.e2. 35985535 10.1016/j.jpeds.2022.08.010 PMC9383958

[4] Naylor R, Phillipson N, *et al*. Diagnosis and clinical management of monogenic diabetes. In: Feingold KRAB, Blackman MR, *et al*., editors. Endotext. South Dartmouth: MDText.com Inc; 2000. 33180404

[5] Malecki MT, Jhala US, Antonellis A, Fields L, Doria A, Orban T, *et al*. Mutations in NEUROD1 are associated with the development of type 2 diabetes mellitus. Nat Genet. 1999;23(3):323–8. 10545951 10.1038/15500

[6] Horikawa Y, Enya M. Genetic dissection and clinical features of MODY6 (NEUROD1-MODY). Curr Diab Rep. 2019;19(3):12. 30793219 10.1007/s11892-019-1130-9

[7] Liu X, Wu C, Li C, Boerwinkle E. dbNSFP v3.0: a one-stop database of functional predictions and annotations for human nonsynonymous and splice-site SNVs. Hum Mutat. 2016;37(3):235–41. 26555599 10.1002/humu.22932 PMC4752381

[8] Lai H, Yang M, Sun M, Pan B, Wang Q, Wang J, *et al*. Risk of incident diabetes after COVID-19 infection: a systematic review and meta-analysis. Metabolism. 2022;137: 155330. 36220361 10.1016/j.metabol.2022.155330 PMC9546784

[9] Rubio-Cabezas O, Minton JA, Kantor I, Williams D, Ellard S, Hattersley AT. Homozygous mutations in NEUROD1 are responsible for a novel syndrome of permanent neonatal diabetes and neurological abnormalities. Diabetes. 2010;59(9):2326–31. 20573748 10.2337/db10-0011 PMC2927956

[10] Brodosi L, Baracco B, Mantovani V, Pironi L. NEUROD1 mutation in an Italian patient with maturity onset diabetes of the young 6: a case report. BMC Endocr Disord. 2021;21(1):202. 34654408 10.1186/s12902-021-00864-w PMC8518322

[11] Horikawa Y, Enya M, Mabe H, Fukushima K, Takubo N, Ohashi M, *et al*. NEUROD1-deficient diabetes (MODY6): identification of the first cases in Japanese and the clinical features. Pediatr Diabetes. 2018;19(2):236–42. 28664602 10.1111/pedi.12553

[12] Dron JS, Wang J, McIntyre AD, Iacocca MA, Robinson JF, Ban MR, *et al*. Six years’ experience with LipidSeq: clinical and research learnings from a hybrid, targeted sequencing panel for dyslipidemias. BMC Med Genom. 2020;13(1):23. 10.1186/s12920-020-0669-2 PMC7011550 32041611

[13] Chang R, Yen-Ting Chen T, Wang S-I, Hung Y-M, Chen H-Y, Wei C-CJ. Risk of autoimmune diseases in patients with COVID-19: a retrospective cohort study. eClinicalMedicine. 2023;56:101783. 36643619 10.1016/j.eclinm.2022.101783 PMC9830133

[14] Montefusco L, Ben Nasr M, D’Addio F, Loretelli C, Rossi A, Pastore I, *et al*. Acute and long-term disruption of glycometabolic control after SARS-CoV-2 infection. Nat Metab. 2021;3(6):774–85. 34035524 10.1038/s42255-021-00407-6 PMC9931026

[15] Yamada S, Motohashi Y, Yanagawa T, Maruyama T, Kasuga A, Hirose H, *et al*. NeuroD/beta2 gene G–>A polymorphism may affect onset pattern of type 1 diabetes in Japanese. Diabetes Care. 2001;24(8):1438–41. 11473083 10.2337/diacare.24.8.1438

[16] Mührer J, Lang-Muritano M, Lehmann R, Blouin JL, Schwitzgebel VM. Atypical familial diabetes associated with a novel NEUROD1 nonsense variant. J Pediatr Endocrinol Metab. 2023;36(1):101–4. 36222545 10.1515/jpem-2022-0356

[17] Bawatneh A, Darwish A, Eideh H, Darwish HM. Identification of gene mutations associated with type 1 diabetes by next-generation sequencing in affected Palestinian families. Front Genet. 2023;14:1292073. 38274107 10.3389/fgene.2023.1292073 PMC10808782

